# Zn-contained mussel-inspired film on Mg alloy for inhibiting bacterial infection and promoting bone regeneration

**DOI:** 10.1093/rb/rbaa044

**Published:** 2020-09-30

**Authors:** Feng Peng, Shi Cheng, Ruiying Zhang, Mei Li, Jielong Zhou, Donghui Wang, Yu Zhang

**Affiliations:** 1 Department of Orthopedics, Guangdong Provincial People's Hospital, Guangdong Academy of Medical Sciences, Guangzhou, Guangdong 510080, China; 2 School of Materials Science and Engineering, Hebei University of Technology, Tianjin 300130, China

**Keywords:** magnesium alloy, zinc, polydopamine, antibacterial, anti-inflammation, osteointegration

## Abstract

Infection and insufficient osteointegration are the main causes of orthopedic implant failure. Furthermore, activating favorable inflammation response is vital to the fast osteointegration of implants. Therefore, endowing the implants with multifunctions (antibacterial, anti-inflammation, and pro-osteointegration) is a promising strategy to improve the performance of orthopedic implants. In this study, a Zn-contained polydopamine (PDA) film was fabricated on AZ31 alloy. The film possessed a stable Zn ion release in 14 days. The results of electrochemical analysis implied the favorable corrosion protection of the film, and thus, leading to a suitable hemolysis ratio (below 1%). The *in vitro* antibacterial assessment revealed that the film exhibited excellent resistance against *Staphylococcus aureus* (nearly 100%), which can be ascribed to the release of Zn ions. The cell-culture evaluation revealed that the extract of Zn-contained PDA-coated sample can activate RAW264.7 polarization to an anti-inflammatory phenotype, as well as enhance the osteogenic differentiation ability of MC3T3-E1. Additionally, the femoral osteomyelitis model indicated that the as-prepared film had a high antibacterial capability at early stage of the implantation, and showed better osteogenesis and osteointegration after 8 weeks of implantation. With favorable antibacterial, anti-inflammation, and pro-osteogenesis abilities, the novel designed Zn-contained PDA film is promising to be used in Mg-based orthopedic implants.

## Introduction

Therapeutic effect of orthopedic implants is closely related to its anti-infection and osteointegration capabilities [1–3]. It is widely believed that 100 bacteria in per gram tissue are enough to result in local infection around implant. The operation site is easily to be infected at initial 3–5 days after surgery [4]. Once bacterial infection occurs, a secondary revision surgery is normally needed to remove bacteria, which increase mental pain and economic stress in patients. In addition, an exceeding immune response would result in a thick fiber layer surrounding the implants, hindering the fast osteointegration of the implant [5]. In addition, long-term clinical statistics revealed that undesirable osteointegration of implants is often observed at late stage of implantation [6–8], which might be owing to the poor bioactive surface of the implants. Hence, antibacterial, faster, and better osteointegration is forever pursuit in the field of orthopedic implants. In addition, biomaterials are experiencing a progress from nondegradable to biodegradable [9–11]. In the past decades, the most extensively used orthopedic implants in clinic are permanent metals such as titanium (Ti), tantalum (Ta), and cobalt–chromium (Co-Cr) alloys [12]. However, the mismatch of elastic modulus between these implants and natural bone would lead to stress shielding. More importantly, once the fracture begins to heal, a second surgery is needed to remove the implant [13–15]. With comparable elastic modulus to natural bone (7–30 GPa) and biodegradable property *in vivo*, magnesium (Mg) and its alloys are regarded as the next generation of biomedical metal implants [16, 17]. To fulfil the requirements (biodegradable, anti-infection, anti-inflammation, and pro-osteogenesis) of next-generation bone implants, it is of great significance to design and fabricate Mg alloys with inhibiting bacterial infection and to promote bone-regeneration abilities.

Surface modification is an effective way to alter biological performances of implant device. Surface properties, including hydrophilicity, roughness, and chemical component, play important roles in cell and bacterial behaviors. For the perspective of antibacterial infections, loaded (or grafted) antibiotics (or antimicrobial peptides) on implant surface is the most effective way to kill bacteria [18, 19]. Yet bacterial resistance is a worldwide concern problem and limits the application of antibiotics. Another promising strategy is immobilization or load of metal ions, e.g. Ag^+^, Cu^2+^, and Zn^2+^ [20]. Ag^+^ and Cu^2+^ would easily cause cytotoxicity, whereas proper Zn^2+^ could inhibit bone resorption and upregulate bone-related gene expression and the activity of alkaline phosphatase (ALP). Furthermore, Zn^2+^ was reported to have anti-inflammatory advantages at a proper concentration, which is beneficial for the tissue-healing process [21, 22]. Hence, Zn^2+^ might be a better choice for the surface modification of orthopedic implants [23–25]. In addition, polydopamine (PDA) gets increasing attention in the field of surface modification for its enhanced osteogenic differentiation ability [26–28]. Polydopamine is formed *via* selfpolymerization of dopamine and contains copious functional groups (–OH, =O, –NH_2_), which is favorable for cell adhesion and can be worked as a platform to chelate metal ions. Therefore, we expected to construct a long-term Zn ion-releasing film on Mg alloy using PDA to inhibit bacterial infection and promote osteointegration.

In this study, the authors first fabricated a hydrothermal film with mixed phase of Mg(OH)_2_ and Mg-Al-layered double hydroxide (Mg-Al LDH) on AZ31 alloy. The previously published studies have proved that such a film can effectively improve corrosion resistance of the substrate [29, 30]. More importantly, its surface was covered by micro–nano plates, and this structure is reported to be favor for cell adhesion and spreading [31–33]. Then PDA films coupled with Zn ions were deposited on hydrothermal-treated AZ31 alloy. Owing to this reason, Zn ions were chelated with PDA during the entire self-polymerization process, Zn ions were evenly distributed across the PDA films, and thus long-term Zn ion releasing can be achieved. The macrophage behaviors influenced by Zn-PDA-coated AZ31 were evaluated. Furthermore, the antibacterial and osteogenesis properties of Zn-loaded PDA film on AZ31 alloy were systematically evaluated *in vitro* and *in vivo*.

## Materials and methods

### Samples fabrication and characterization

AZ31 alloys were cut into dimensions of ∅ 10 mm×2 mm (for *in vitro* tests) and ∅ 2 mm×10 mm (for *in vivo* tests), ground with 1000# SiC paper, ultrasonically cleaned in ethyl alcohol, and dried in air. The cut AZ31 alloys were put in a Teflon-lined stainless 50 ml of Al(NO_3_)_3_ solution was added (0.02 M, pH value adjusted to 12.8 with NaOH). After being reacted at 120°C for 12 h, the samples were taken out, cleaned with deionized water, and denoted as LDH#. To prepare PDA-coated samples, 50 mg of dopamine–HCl (Aladdin, USA) was dissolved in 10 ml of Tris–HCl solution (10 mM, pH = 8.5) and then LDH# samples were immersed in the dopamine solution and protected from light. After reacted at 37°C for 12 h, the obtained samples were denoted as LDH/PDA#. To prepare Zn-containing PDA-coated samples, Zn(NO_3_)_2_ was added into dopamine solution with concentrations of 1, 2, and 5 mg/mL, and LDH# samples were immersed in these dopamine solutions for 12 h at 37°C. The obtained samples were denoted as Zn-1#, Zn-2#, and Zn-3#.

The surface views of AZ31, LDH#, LDH/PDA#, Zn-1#, Zn-2#, and Zn-3# samples were observed by using scanning electron microscopy (SEM; Hitachi-S3400N, Hitachi, Japan). The phase compositions and chemical element compositions of all the samples were detected using X-ray diffraction (XRD; D/Max, RIGAKU, Tokyo, Japan) and X-ray photoelectron spectroscopy (XPS; PHI 5802, Physical Electronics Inc., Eden Prairie, MN, USA), respectively.

### Mg and Zn ion release

The samples were immersed in 10 ml of Phosphate-buffered saline (PBS) at 37°C. At day 1, 4, 7, 10, and 14, the extracts were collected and added with another 10 ml of PBS. The amount of Mg ions and Zn ions in the extracts was determined by using an inductively coupled plasma/optical emission spectroscopy (ICP-OES; Vista AX, Varian, USA).

### Electrochemical analysis

The electrochemical analysis was applied on a CHI760C electrochemical analyzer (Shanghai, China) with a three-electrode electrochemical cell. Phosphate-buffered saline was used as electrolyte and the exposure area of the sample to electrolyte was 0.255 cm^2^. The samples were stabilized in PBS to get a stable open-circuit potential prior to the test. Potentiodynamic polarization (PDP) curve was performed with a sweep rate of 10 mV/s. The corrosion potential (*E*_corr_), current density (*j*_corr_), and corrosion resistance (*R_p_*) were determined by Tafel extrapolation. The surface views of the samples after PDP test were observed using SEM.

Electrochemical impedance spectroscopy (EIS) measurement was also applied on the same electrochemical analyzer. The test was conducted with a sinusoidal perturbing signal of 5 mV and recorded from 100 kHz to 10 mHz. The results were analyzed on ZView software.

### Hemolysis ratio assay

Fresh whole blood, donated by a healthy volunteer, was diluted with 0.9 wt% NaCl at a volume ratio of 4:5. Distilled water and 0.9 wt% NaCl were used as positive and negative controls, respectively. The samples were placed in a 24-well plate and then 2 ml of 0.9 wt% NaCl was added into the well. After being kept at 37°C for 30 min, 40 μL of diluted blood was added into the plate and incubated for another 60 min. The solutions were collected and centrifuged at 3000 rpm for 5 min. The absorbance (545 nm) of the supernatant was measured using an enzyme labeling instrument (BIO-TEK, ELX 800). The hemolysis rate was calculated *via* the equation:

$$\text{Hemolysis}\;\text{ratio}=\left({\text{AS}}_{545}-{\text{AN}}_{545}\right)/\left({\text{AP}}_{545}-{\text{AN}}_{545}\right)\times 100\%$$

Where AS_545_, AN_545_, and AP_545_ are the measured value of the samples, negative control, and positive control, respectively.

### *In vitro* antibacterial evaluation

#### Live/dead staining and SEM observation

Gram-positive *Staphylococcus aureus* (*S. aureus*, ATCC 25923) was used to investigate the antibacterial ability of various samples. The samples were sterilized by exposing to ultraviolet for overnight. In brief, 800 μL of bacterial suspension (10^7^ CFU/mL) was seeded on the samples and incubated for 1 day. Then, the samples were rinsed with 0.9 wt% NaCl and 100 μL fluorescence dying solution (5 μM propidium iodide and 2 μM calcium–AM) was added into each sample. After being cultured for another 15 min, the samples were washed with 0.9 wt% NaCl and observed using confocal laser scanning microscopy (CLSM; Leica SP8, Germany). In addition, *S. aureus* were seeded on the samples in the same method and cultured for 1 day. Afterward, bacteria were fixed in 2.5 v% glutaraldehyde, dehydrated in ethanol, and dried in hexamethyldisilizane ethanol solution. The bacterial morphologies were then observed.

#### Recultivated colony and inhibition zone observation

In total, 800 μL *S. aureus* suspension (10^7^ CFU/mL) was seeded on the samples and incubated for 1 day. Then, the samples were transferred to tubes (4 ml 0.9 wt% NaCl in each tube) and the bacteria were detached from the sample by mechanical shaking. After diluting 10 times with 0.9 wt% NaCl, 100 μL of diluted bacterial suspension was inoculated into tryptic soy broth (TSB) agar culture medium and spread out the bacteria evenly with a push rod. After being cultured for another 18 h, the bacteria were pictured using a gel imaging analysis system (GIA; FluorChem M, Protein Simple). To observe the inhibition zone, 100 μL of *S. aureus* suspension (10^7^ CFU/mL) was spread out evenly on TSB agar culture medium and the samples were placed on its surface. After being cultured for 18 h, the bacteria were pictured using a GIA system.

### Cell culture

The murine-derived macrophage cell line RAW264.7 cells and mouse osteoblast cell line MC3T3-E1 were purchased from Cell Bank of the Chinese Academy of Sciences. MC3T3-E1 and rBSMCs were cultured in α-MEM supply with 10% of fetal bovine serum (Gibco, Tauranga, New Zealand) and 1% of penicillin and streptomycin (P/S); RAW264.7 was cultured in the Dulbecco’s modified Eagle medium: nutrient mixture F-12 (DMEM/F12, Hyclone) was supplemented with 10% of fetal bovine serum and 1% of P/S. The extracts were used to evaluate the biological effects of various samples. The sterilized samples were immersed in corresponding culture medium (a volume ratio of 1.25 cm^2^/mL) and then placed in incubator for 1 day. The extracts mentioned below were harvested in this method.

### Cytocompatibility evaluation

#### Cell adhesion

MC3T3-E1 were seeded on various samples at 5 × 10^4^ cells/mL in 24-well plates. The cells were cultured for 1, 4, and 24 h, and were then rinsed by PBS and fixed in 4% paraformaldehyde. After that, the cells were successively permeabilized by Triton X-100, blocked by bovine serum protein. Then F-actin was stained with FITC phalloidin and nucleus was stained with DAPI. Finally, the stained cells were observed using CLSM (Leica SP8, German).

#### Live/dead staining

MC3T3-E1 were seeded on various samples at 5 × 10^4^ cells/mL in 24-well plates for 7 days. At the scheduled time, the cells were rinsed by PBS, and then added with 100 μL of dying solution (propidium iodide, 5 μM and calcium-AM, 2 μM; Sigma, USA). After being stained for 15 min, the samples were rinsed by PBS and kept in PBS. The stained cells were observed by using CLSM.

### Polarization of RAW264.7

The polarization of RAW264.7 was evaluated using quantitative real-time PCR analysis (qRT-PCR). The Raw264.7 were seeded in 12-well plate with a concentration of 5 × 10^4^ cells/well. After being cultured for 1 day, the culture mediums were replaced with various extracts. Further culturing for 3 days, the total RNA of Raw264.7 was purified by the TRIzol reagent (Invitrogen). NanoDrop spectrophotometer (Thermo Fisher Scientific, USA) was adopted to measure the concentration of RNA. First, standard complementary DNA (cDNA) was synthesized based on total RNA using the first-strand cDNA synthesis supermix Kit (Yeasen, China). Synthesized cDNA was then mixed with SYBR Green Mastermix and primers and running RT-PCR programs on a CFX Connect Real-Time PCR Detection System (BIO-RAD, USA). Data were analyzed according to 2^−ΔΔ^CT method. The primer sequences [tumor necrosis factor-α (TNF-α), interleukin-1β (IL-1β), interleukin-6 (IL-6), interleukin-10 (IL-10), CD163, and chemokine ligand-3 (CCL-3)] are listed in Table 1.

**Table 1.** Primer sequences used in qRT-PCR

Gene	Forward primer sequence(5′-3′)	Reverse primer sequence(3′-5′)
TNF-α	GGACTAGCCAGGAGGGAGAA	CGCGGATCATGCTTTCTGTG
IL-1β	CCCAACTGGTACATCAGCACCTC	GACACGGATTCCATGGTGAAGTC
IL-6	CTGCAAGAGACTTCCATCCAG	AGTGGTATAGACAGGTCTGTTGG
IL-10	GCTCTTACTGACTGGCATGAG	CGCAGCTCTAGGAGCATGTG
CD163	ATGGGTGGACACAGAATGGTT	CAGGAGCGTTAGTGACAGCAG
CCL-3	TGTACCATGACACTCTGCAAC	CAACGATGAATTGGCGTGGAA
ALP	ACTCAGGGCAATGAGGTCAC	CACCCGAGTGGTAGTCACAA
RUNX-2	GTGGCAGTGTCATCATCTGAAAT	TCGCCTCAGTGATTTAGGGCGCA
COL-I	CCTAATGCTGCCTTTTCTGC	ATGTCCCAGCAGGATTTGAG
OCN	AGACTCCGGCGCTACCTT	CTCGTCACAAGCAGGGTTAAG
GAPDH	TGTCCGTCGTGGATCTGAC	CCTGCTTCACCACCTTCTTG

### Osteogenic ability of MC3T3-E1

#### Alkaline phosphatase activity assay

MC3T3-E1 cells were seeded in 24-well plates with a concentration of 1 × 10^5^ cells/well for 1 day. The culture medium was changed with various extracts (supplied with 10 mM β-glycerophosphate, 100 nM dexamethasone, 50 mM ascorbate, and glutamine to induce differentiation) at day 2 and further cultured for 7 and 14 days. The staining of ALP was performed by using BCIP/NBT ALP color development kit (Beyotime, China). Quantitative analysis of ALP activity was performed by ALP kit (Beyotime, China). Both the qualitative and the quantitative assays were performed according to the manufacturer’s instructions.

#### QRT-PCR evaluation

MC3T3-E1 were seeded at 2 × 10^5^ cells/mL in 6-well plates. The culture medium was replaced by various extracts after being cultured for 1 day. After being cultured for 7 and 14 days, the expression of osteogenesis-related genes [ALP, Collagen-I (Col-I), osteocalcin (OCN), and Runt-related transcription factor-2 (RUNX-2)] was detected by qRT-PCR technology as mentioned in *Cytocompatibility evaluation* section. The involved primer sequences are listed in Table 1.

### Animal experiment

All the procedures of animal experiment were approved by the Animal Ethics Committee of Guangdong Provincial People’s Hospital and applied in accordance with the Guidelines for Care and Use of Laboratory Animals of Southern Medical University.

#### Surgical procedure for bone implantation

Sixteen male Sprague–Dawley (SD) rats (age, 3 months; weight, 200–300 g) were used to establish a femoral osteomyelitis model. Before surgery, rats were subjected to intraperitoneal injection of pentobarbital sodium (40 mg/kg) to anesthetization. Both legs were shaved and sterilized by iodine tincture. A lateral longitudinal skin incision was made on the side of the patella and the patellofemoral groove of the distal femur was exposed. Afterward, a tunnel with a diameter of 2 mm and 30 mm in length was drilled into the medullary cavity of femur along the axis of the femoral shaft with rinsing of 0.9% of saline. In brief, 20 μL of *S. aureus* suspension (10^4^ cfu/mL) was injected into the femur. The sterilized rod samples were carefully inserted into the femur canal, and the fascia and skin were gently sutured and covered with sterile gauze.

#### Antibacterial assessment

After implantation for 3 days, eight rats (two rats per group) were sacrificed. The implants were carefully taken out from the femurs and rolled across the sterilized TSB agar plates. After being cultured for another 18 h, the agar plates were pictured by using a GIA system. In addition, the remaining femurs were fixed in paraformaldehyde, dehydrated, embedded in paraffin, and stained with hematoxylin and eosin (H&E).

#### Micro-CT evaluation

At 8 weeks postsurgery, eight rats were sacrificed and the femurs containing the specimens were scanned by micro-CT (Aloka Latheta LCT-200, HITACHI, Japan) at a resolution of 48 mm under 80 kV and 40 μA. Representative 2D radiographic images were reconstructed into three-dimensional (3D) images by Multimodal 3D Visualization (Siemens, Germany) software. The bone volume percentage within a region of interest and the bone mineral density, termed BV/TV and BMD, respectively, were also analyzed.

#### Histological examination

After micro-CT scanning, the femur specimens were dehydrated by gradient ethanol and embedded in poly(methyl methacrylate). The specimens were cut into hard tissue sections and further polished and grinded into 40–50μm thickness sections by using the cutting grinding system (EXAKT Apparatebau, Norderstedt, Hamburg, Germany). The tissue sections were stained with van Geison’s (VG) solution and imaged under a microscope (Olympus, Germany).

### Statistical analysis

Data were expressed as the mean ± standard deviation. Differences among groups were analyzed with two-way ANOVA followed by the Tukey’s *post-hoc* test using GraphPad Instant software. *P* < 0.05 was considered a significant difference.

## Results and discussion

### Film characterization and corrosion resistance evaluation

**Figure 1.**
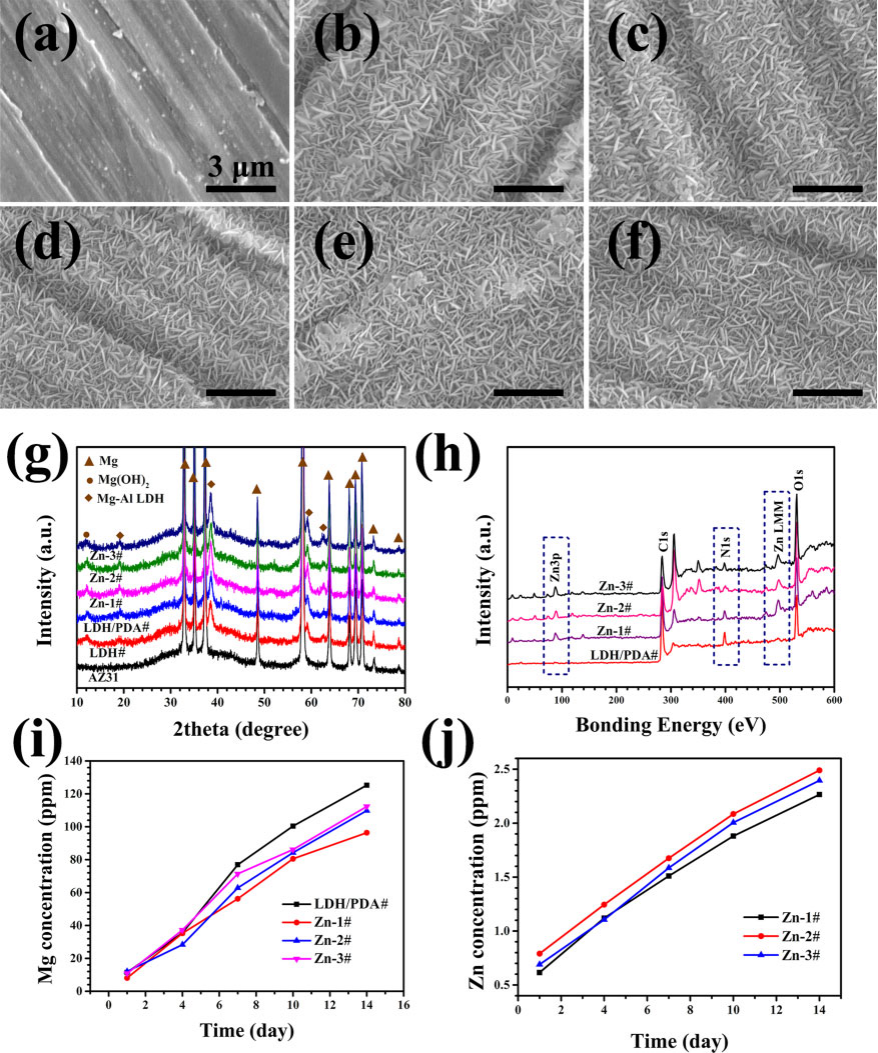
Surface views of AZ31 (a), LDH# (b), LDH/PDA# (c), Zn-1# (d), Zn-2# (e), and Zn-3# (f) samples, and corresponding XRD patterns (g). XPS full spectra of PDA coated samples (h). Cumulative profiles of Mg ion (i) and Zn ion (j) release from Zn-contained PDA-coated samples in PBS

Surface morphologies of various samples are shown in Fig. 1a–f. Obvious grounding traces could be observed on the surface of AZ31 (Fig. 1a). After being hydrothermally treated and further immersed in dopamine solution, the grounding traces remained. The surface of LDH# sample was covered by a compact plate-like structure (Fig. 1b), a typical morphology of Mg(OH)_2_ and LDH [34–36]. Further coated with PDA (Fig. 1c) and Zn-contained PDA films (Fig. 1d–f), the surface morphologies remained unchanged. This is because PDA films were uniformly deposited on the samples’ surface and their thickness are between 20 and 50 nm [37, 38]. Figure 1g shows the XRD patterns of various samples. Only Mg phases were detected on the surface of AZ31. For the pattern of LDH# sample, the peaks at 12 and 18° indicated the formation of Mg(OH)_2_ and Mg-Al LDH phases, respectively. The XRD patterns of PDA-coated samples were similar with that of LDH# sample. The element compositions of PDA-coated samples were analyzed by XPS. As shown in Fig. 1h, N 1s feature peak was appeared on all PDA-coated samples, indicating the successful deposition of PDA film. Furthermore, Zn 3p feature peak was appeared only on the spectrum of Zn-1#, Zn-2#, and Zn-3# groups, suggesting the successful anchor of Zn ions in PDA film. The semiquantitative results of Zn and N contents by XPS are summarized in Table 2. The thickness of Zn-contained PDA films was thinner than PDA film, which was verified by the decrease of N content. The content of Zn in the surface of Zn-contained PDA films ranged from 1.81 to 2.58 at%. It was widely reported that metal ions can be chelated with phenolic groups [39, 40], whereas a dopamine molecule contains two phenolic hydroxyl groups. Therefore, the Zn ions can easy be chelated in PDA film. However, because Zn ions occupied the reactive sites of dopamine, the selfpolymerization process of dopamine was suppressed in some degree, leading to a thinner PDA film. Figure 1i and j shows the cumulative release of Mg and Zn ions, respectively. It can be observed that the appearance of Zn ions had little influence in the release of Mg ions. In addition, the release of Zn ions of the three Zn-contained PDA films was in the similar level. Because there were few differences of the surface morphology, phase compositions, Mg and Zn ions release between the three Zn-containing PDA films, their performances in corrosion resistance and biological effects are believed to be similar with each other. Hence, the authors choose Zn-2# sample as the typical Zn-contained PDA film to evaluate their *in vitro* and *in vivo* behaviors.

**Table 2.** Content of Zn and N elements in LDH/PDA#, Zn-1#, Zn-2#, and Zn-3# samples

	LDH/PDA#	Zn-1#	Zn-2#	Zn-3#
Zn (at%)	0	2.57	1.81	2.46
*N* (at%)	7.25	5.59	3.95	4.35

**Figure 2.**
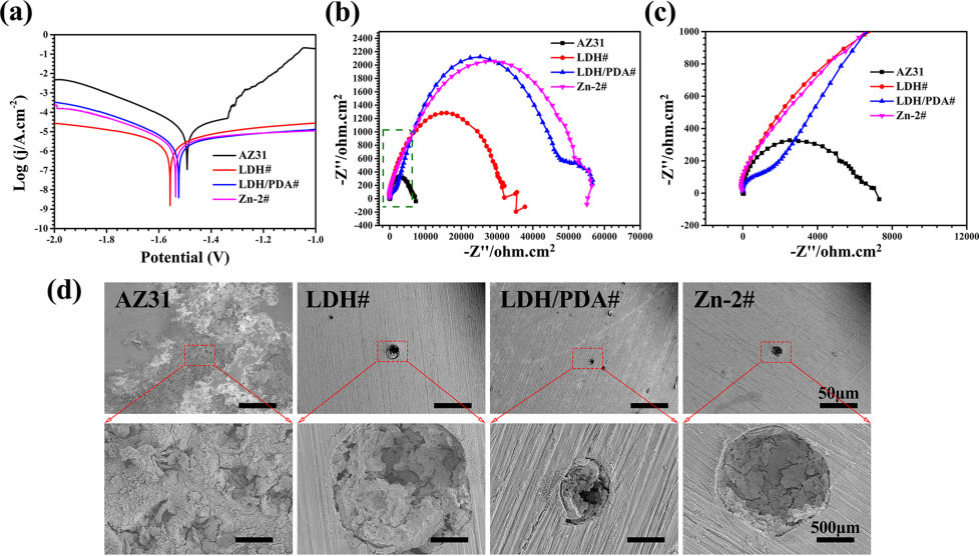
Potentiodynamic polarization (a) and EIS (b) results of AZ31, LDH#, LDH/PDA#, and Zn-2# samples. The magnification of the block in (b) is shown in (c). Surface images of AZ31, LDH#, LDH/PDA#, and Zn-2# samples after PDP test (d).

Fast degradation of Mg-based implants will result in their mechanical failure, and the byproducts, such as superfluous hydrogen and hydroxyl, will lead to inflammation response and poor osteointegration. Therefore, corrosion resistance is the first primary consideration for the clinical applications of Mg-based implants. The corrosion resistance of various samples was evaluated by electrochemical analysis. Figure 2a shows the PDP curves and the corresponding calculated data are listed in Table 3. The *E*_corr_ values of all the surface modified samples were slightly lower than that of untreated AZ31 sample. During the process of the electrochemical test, a corrosion product, mainly composed of Mg(OH)_2_, will form on the surface of bare AZ31 substrate, leading to a relative low *E*_corr_ value, which was also reported by other researchers [40]. However, the *j*_corr_ values of all the surface-modified samples were about 10 orders lower than that of untreated AZ31 sample, indicating the enhanced corrosion resistance of LDH#, LDH/PDA# and Zn-2# samples. The conclusion was also certified by the calculated *R_p_* values. Notably, LDH/PDA# and Zn-2# film exhibited lower *j*_corr_ values and higher *R_p_* values than LDH# group, revealing the improved protection of AZ31 substrate after PDA was coated. The SEM images of all the samples after PDP test are shown in Fig. 1d. A large scale of electro-corrosion area was observed on the surface of AZ31. However, corrosion pits were observed on the surface of LDH#, LDH/PDA#, and Zn-2# groups, and the diameters of corrosion pits were 189 ± 3 , 77 ± 12, and 145 ± 8 μm, respectively. This result also proved that PDA-coated samples showed better corrosion resistance than AZ31 and LDH# samples. The spectra of EIS are shown in Fig. 2b and c. LDH/PDA and Zn-2# samples showed remarkably larger impedance than AZ31 and LDH# samples. The fitted electrical circuit can be illustrated as *R_s_* (*Q_f_* (*R_f_* (*Q_dl_ R_ct_*))) (Supplementary Fig. S1). *R_ct_* means the reaction resistance and the fitted values are listed in Table 2. The *R_ct_* values of LDH/PDA and Zn-2# samples were about 5 and 1.3 orders of AZ31 and LDH# samples, respectively. The EIS results were consistent with the results of PDP.

**Table 3.** Corrosion data of AZ31, LDH#, LDH/PDA#, and Zn-2# samples

	*E*_corr_ (V)	*j*_corr_ (A/cm^2^)	*R_p_* (Ω/cm^2^)	*R*_ct_ (Ω/cm^2^)
AZ31	−1.50	1.22 × 10^−5^	1.15 × 10^5^	1304
LDH#	−1.56	1.42 × 10^−6^	8.82 × 10^5^	4770
LDH/PDA#	−1.52	1.60 × 10^−6^	9.40 × 10^5^	6469
Zn-2#	−1.54	1.60 × 10^−6^	9.43 × 10^5^	6576

Many studies have proved that Mg(OH)_2_ and Mg-Al LDH composite film can enhance the corrosion resistance of Mg alloy [29, 41, 42]. However, to fulfil the requirements of orthopedic applications, it is essential to endow the surface with more functions. In this study, dopamine is chosen to introduce Zn into the surface and further improve its corrosion resistance. Dopamine, a part of human neurotransmitter system, belongs to catecholamine and phenethylamine families. It could quickly selfpolymerize to PDA in basic aqueous solution and the PDA layer could almost be formed on all the material’s surface. Thanks to the sufficiently impermeable ability of PDA molecule, a thin PDA film can significantly prevent the permeation of corrosive medium, and thus lead to a more enhanced corrosion resistance [28, 43].

### Hemolysis ratio and cytocompatibility evaluation

**Figure 3.**
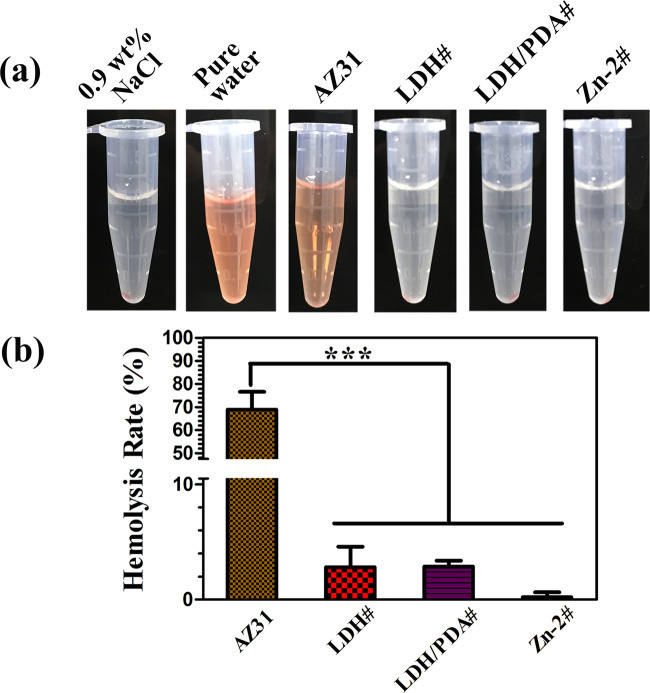
Optical images of collected extracts after centrifugation (a). Hemolysis ratio of AZ31, LDH#, LDH/PDA#, and Zn-2# (b).

For implanted materials, hemolysis ratio is a key factor to evaluate damage to erythrocyte from materials. As shown in Fig. 3a, the extract of negative control and AZ31 groups were red, indicating the rupture of erythrocyte membrane. However, for positive control and surface-treated groups, precipitations were observed on the bottom of the tubes, suggesting that erythrocyte kept well after being incubated in these samples. The quantitative results of hemolysis ratio are shown in Fig. 3b. The hemolysis ratio of AZ31 sample was almost 70%, far higher than the requirement of clinical applications (below 5%). The hemolysis ratios of LDH#, LDH/PDA#, and Zn-2# samples were all below 4%, which were suitable for clinical applications. The LDH film on AZ31 alloy can significantly enhance its corrosion resistance, thus resulting in a moderate change of pH value and Mg ion concentration of the surrounding medium. Hence, LDH-based films showed few damages to erythrocyte.

**Figure 4.**
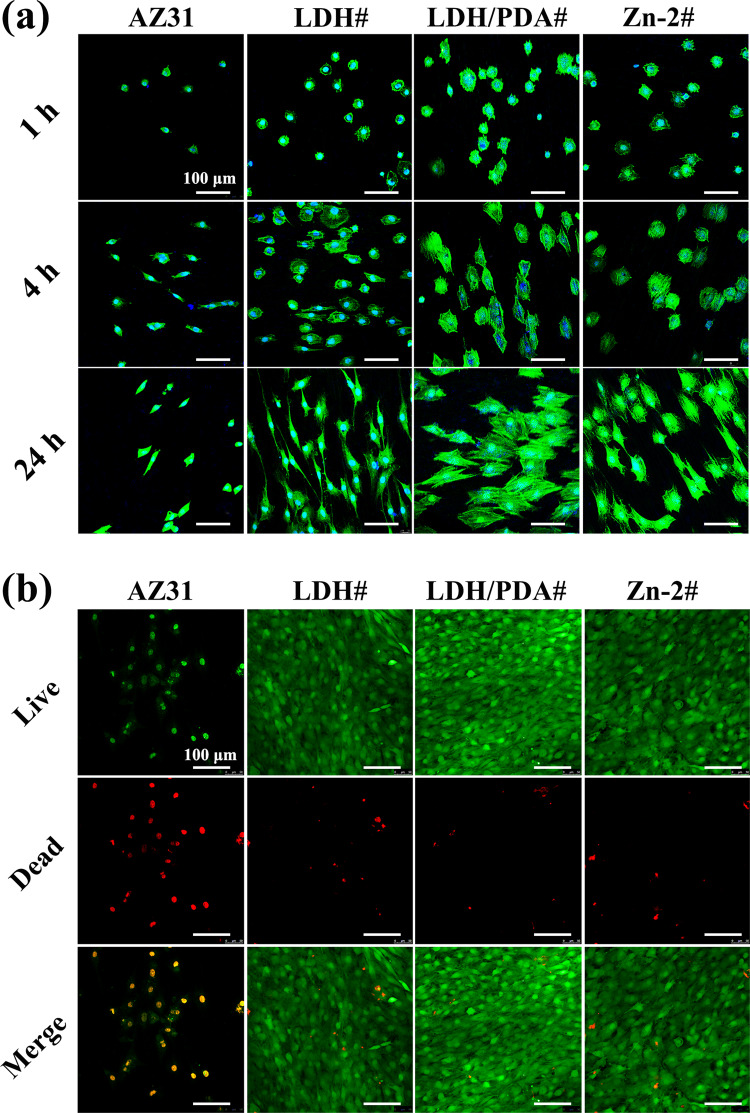
Skeleton staining of MC3T3-E1 after being cultured on LDH#, LDH/PDA#, and Zn-2# samples for 1, 4, and 24 h (a); blue indicates nucleus and green indicates actin. Live/dead staining images of MC3T3-E1 after being cultured on LDH#, LDH/PDA#, and Zn-2# samples for 7 days (b); green indicates living cells and red indicates dead cells.

To study *in vitro* cytocompatibility of the samples, both early cell adhesion and live/dead staining were applied on MC3T3-E1. Figure 4a shows the skeleton stain images of MC3T3-E1 cultured on various samples’ surface. After being cultured for 1 h, a larger number of cells were observed on the three coated samples than on AZ31. The cells in all the samples showed a round morphology. But the cells cultured in the surface of LDH/PDA# and Zn-2# samples showed a significantly larger spreading area than the cells in AZ31 and LDH# samples. After 4 h of incubation, the spreading area increased for all the cells, and the trend of spreading area remained. When the culture duration extended to 24 h, the cells in the AZ31 surface still displayed a hardly spreading morphology, whereas the cells in the LDH# surface exhibited a long strip morphology. For the cells cultured in LDH/PDA and Zn-2# surfaces, many lamellipodia were observed, and an obviously larger spreading area than those cells in AZ31 and LDH# surfaces. Notably, at all the detected points of time, the spreading of cells in Zn-2# surface was poor than in LDH/PDA# surface, but better than in AZ31 and LDH# surfaces. The above data revealed that LDH# surface could support for cell adhesion and spreading, which was owing to the corrosion resistance of Mg-Al LDH film. Further coated with PDA on LDH film, its corrosion resistance was further improved, and many bioactive groups (–NH_2_, –OH, and =O) were introduced on the surface. Thus, cells showed an even more spreading morphology on LDH/PDA# surface. For Zn-2# sample, the bioactive groups on PDA film decreased, leading to a decrease in cells’ adhesion and spreading. Figure 4b shows the results of live/dead staining. Only dead cells were observed on the surface of AZ31. For all the three coated samples, only a few dead cells were observed, and a large number of living cells covered on the samples’ surface. This result indicated that cells could survive and proliferate on LDH-coated sample, and further modified with PDA or Zn-contained PDA would not do harm to cells’ viability. The good cytocompatibility of the three coated samples was also verified via culturing MC3T3-E1 in their extracts (Supplementary Fig. S2). Cells cultured in the extracts of the three coated samples exhibited an increased proliferation over time, whereas a relative low viability was observed when cultured in the extract of AZ31.

In all, the data of hemolysis ratio, early adhesion, and live/dead staining verified that Zn-2# sample possesses good biocompatibility.

### Antibacterial and anti-inflammatory properties

**Figure 5.**
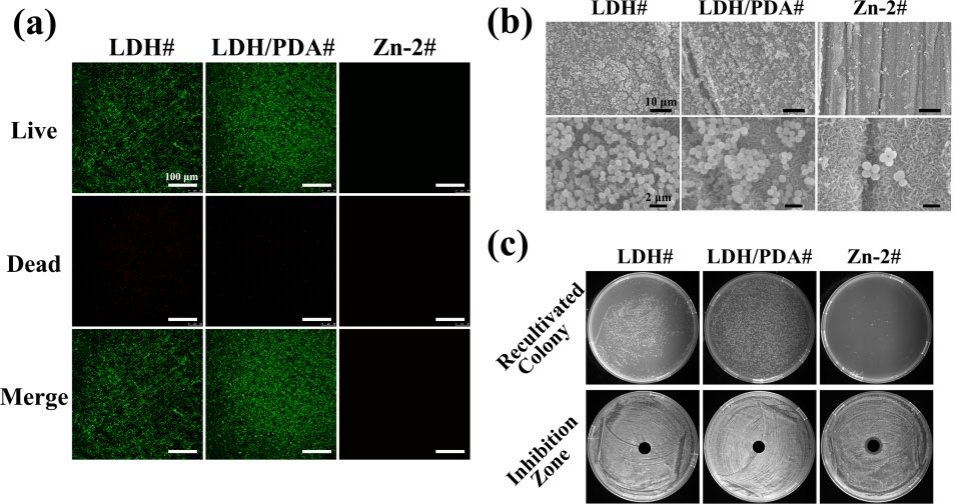
Live/dead staining images (a) and SEM views (b) of *S. aureus* after being cultured on LDH#, LDH/PDA#, and Zn-2# samples. Recultivated colony of *S. aureus* cultured on various samples and inhibition zone surrounding various samples against *S. aureus* (c).

Infection is one of the main causes for the failure of the implantation. Figure 5a shows the live/dead staining of *S. aureus* after being cultured on various samples for 1 day. Many living bacteria were observed on the surface of LDH# and LDH/PDA# samples, whereas almost no living bacteria were detected on the surface of Zn-2# sample. Furthermore, the bacteria were observed using SEM (Fig. 5b). It can be observed that a large number of bacteria gather together on LDH# and LDH/PDA# surfaces. On the surface of Zn-2#, a few *S. aureus* were observed, and only several bacteria were gather together, suggesting that it was hard to form bacterial film. On all the samples’ surface, the shapes of *S. aureus* were round and no destroy of cell membrane was observed. These results demonstrated that Zn-2# sample can inhibit the proliferation of bacteria. Furthermore, bacteria were detached from the samples and recultured in agar plats (Fig. 5c). For LDH# and LDH/PDA# samples, many bacterial colonies grew on the agar plate, whereas almost no bacterial colony was observed for the Zn-2# sample. This result suggested a nearly 100% antibacterial capacity of Zn-2# sample. Figure 4c also shows the images of inhibition zones of various groups. An obvious antibacterial ring appeared on Zn-2# group, demonstrating that the release of Zn ions from Zn-2# sample was responsible for its antibacterial ability. The desirable antibacterial capability of Zn ions has been proved by numerous studies. There is a study which revealed that Zn ions possessed strong antibacterial *via* inducing excess reactive oxygen [44]. In addition, there are hypotheses claimed that positive Zn ions could adhere to the negatively charged bacteria membrane, and result in the membrane dysfunction. In our study, no disruption of bacteria membrane was observed, thus, an excessive production of reactive oxygen induced by Zn ions might be used to explain the antibacterial capability of Zn-2# sample.

**Figure 6.**
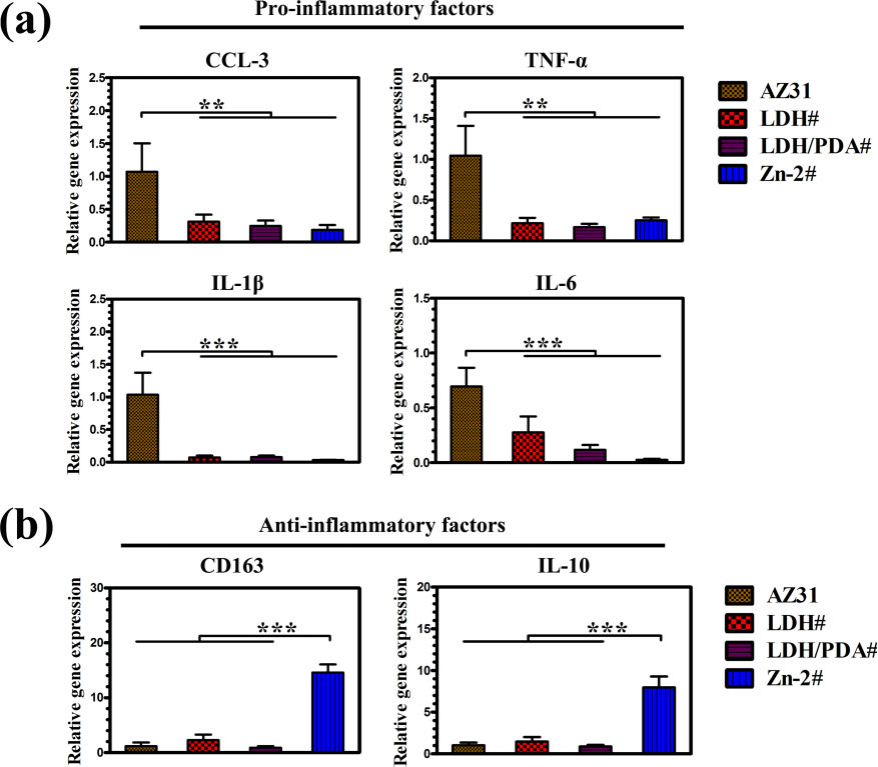
Gene expression of proinflammatory (a) and anti-inflammatory (b) factors by RAW264.7 cells cultured in the extracts of AZ31, LDH#, LDH/PDA#, and Zn-2#.

A series of signaling molecular cytokines, and receptor released by immune cells would take part in the bone remodeling process [5, 45]. Macrophage plays a central role in immune response. When the biomaterials are implanted, macrophage will activate to M1 phenotype (proinflammatory) or M2 phenotype (anti-inflammatory). The fiber promoted by M1 phenotype will inhibit the fast osteointegration between the implant and the bone, whereas anti-inflammatory cytokines and growth factor released by M2 phenotype can promote the growth of new bone. In this study, RAW264.7 was cultured in various extracts to evaluate their immune response. Cells in the extracts of coated samples, especially for the extract of Zn-2# sample, exhibited a significant higher proliferation than bare AZ31 (Supplementary Fig. S3), indicating the good cytocompatibility of the coated samples to RAW264.7. Figure 6a and b shows the M1-related and M2-related gene expression of RAW264.7, respectively. It can be observed that cells cultured in the extracts of LDH#, LDH/PDA#, and Zn-2# exhibited significantly lower gene expression of CCL-3, TNF-α, IL-1β, and IL-6. For anti-inflammatory factor (CD163 and IL-10), cells cultured in Zn-2# samples’ extract exhibited a higher expression than the other three extracts. The above data revealed that Zn-2# sample was more favorable for RAW264.7 polarizing to M2 phenotype. This result was consistent with the conclusions of the report by Liu *et al.* [46]. In their study, they used magnetron sputtering to fabricate a Zn layer on sulfonated polyetheretherketone and found that such a Zn layer can guide macrophage polarize to M2 phenotype and induce the secretion of anti-inflammatory and osteogenic cytokines. More importantly, through the detection of whole mRNA expression, they found that the NF-κB signaling pathway and the Jak-STAT signaling pathway were responsible for the M1 phenotype-related pathway and M2 phenotype-related pathway, respectively.

### Osteogenic differentiation evaluation

**Figure 7.**
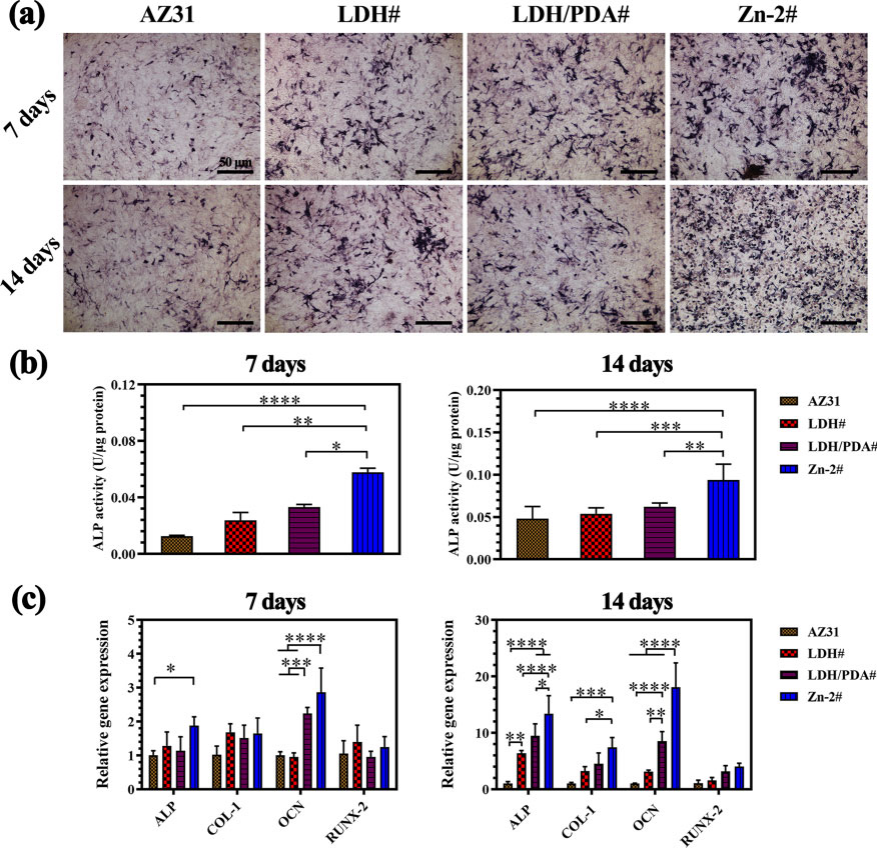
Images of ALP staining after osteogenic induction for 7 and 14 days (a), and corresponding quantitative results (b). Osteogenesis-related gene expression after osteogenic induction for 7 and 14 days (c).

The activity of ALP is one of the most vital signs for osteogenic differentiation. Figure 7a shows the stained ALP of MC3T3-E1 after being cultured in various extracts for 7 and 14 days, and the corresponding quantitative analyses are shown in Fig. 7b. Both at day 7 and 14, cells exhibited a highest ALP activity when cultured in the extract of Zn-2# sample. Although at day 7, cells cultured in the extracts of LDH# and LDH/PDA# samples showed a higher ALP activity than in the extract of AZ31, but no significant differences were observed. The osteogenic differentiation influence of various samples on MC3T3-E1 was also evaluated on the molecular level by using qRT-PCR technology. At day 7, no differences were observed on the expression of RUNX-2 and COL-I genes between the cells cultured in all the four extracts (Fig. 7c). For the gene of ALP, the cells cultured in the extract of Zn-2# exhibited a significantly higher expression than in that of AZ31, but no difference with LDH# and LDH/PDA# groups. For the gene of OCN, cells in the extract of LDH/PDA and Zn-2# samples showed a higher expression than in the other two extracts. When culture duration extended to 14 days, still no difference was observed for RUNX-2 gene between the four groups. However, for the ALP, COL-I, and OCN genes, the cells showed a higher expression for Zn-2# group. The highest ALP activity and expression of osteogenesis-related genes suggested the favorable osteogenic induction of Zn-2# sample. The osteogenesis induction of Zn ions has been widely studied, and many researchers used Zn to modify the implant surface or manufacture novel Zn-contained alloys/bio-ceramics [47–49]. Notably, Huo et al. [50] fabricated a titania nanotube incorporated with Zn on Ti surface, and demonstrated that ERK1/2 signaling pathway was involved in the enhanced osteogenesis process.

### *In vivo* evaluation

**Figure 8.**
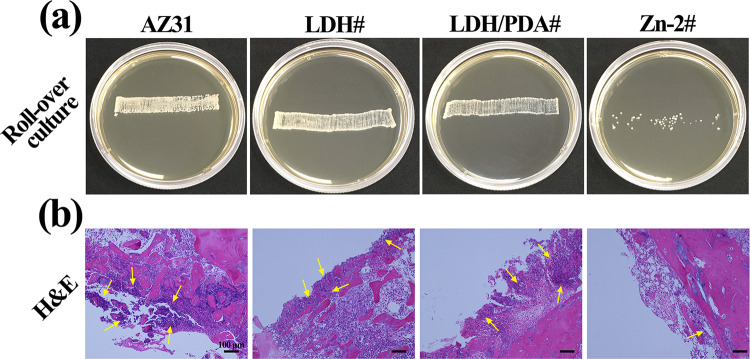
Recultivated colony of *S. aureus* from various samples after being implanted for 3 days with bacterial infection (a), and corresponding H&E staining images of the femurs (b); the yellow arrows indicate neutrophils.

To study the antibacterial, anti-inflammation, and bone regeneration properties of the samples, an implantation test was applied in the rat femurs with the presence of *S. aureus*. For the study of *in vivo* antibacterial property of Zn-contained PDA film, the implants were taken out after implantation for 3 days, and rolled across the sterilized TSB agar plates. As shown in Fig. 8a, it is clear that a large number of bacterial colonies reproduced for AZ31, LDH#, and LDH/PDA# groups. However, for Zn-2# sample, only a few bacteria grew colonies, suggesting its favorable *in vivo* antibacterial. Pure Mg and Mg alloys were reported to have strong antibacterial ability *in vitro* [51, 52]. However, their antibacterial *in vivo* was not as good as *in vitro* [52], which might be because they would experience a decreased corrosion rate *in vivo* and the complex *in vivo* microenvironment was more favorable for cell adhesion and proliferate. In this study, we also found that AZ31 alloy showed an undesirable antibacterial ability *in vivo*. However, after coated with Zn-contained PDA film, the sample exhibited a sufficient antibacterial property, indicating its potential applications in the anti-infection of the implant. In addition, to study the *in vivo* anti-inflammatory ability of the samples, the H&E staining was applied after the samples were implanted for 3 days (Fig. 8b). Compared with AZ31, LDH#, and LDH/PDA# groups, less neutrophils (indicated by yellow arrows) were observed on Zn-2# group, suggesting its mildest inflammatory response.

**Figure 9.**
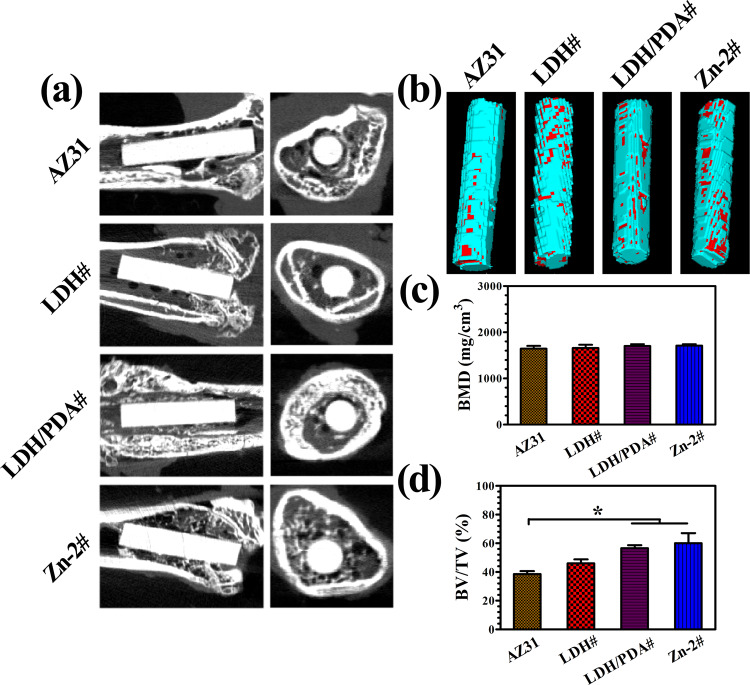
Micro-CT analysis of AZ31, LDH#, LDH/PDA#, and Zn-2# samples after femur implantation for 8 weeks (a), and corresponding 3D reconstruction images of micro-CT results (b); red sections indicate new bone. Calculated bone volume/tissue volume (BV/TV) (c) and trabecular bone mineral density (BMD) (d).

**Figure 10.**
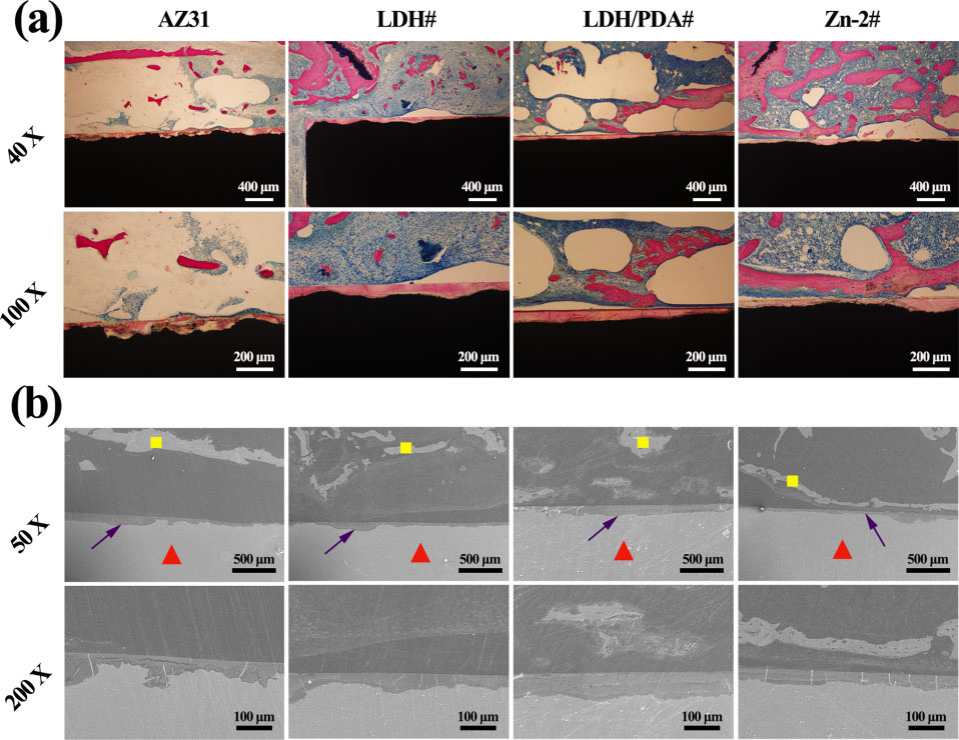
VG staining of AZ31, LDH#, LDH/PDA#, and Zn-2# samples after femur implantation for 8 weeks (a). SEM observations of AZ31, LDH#, LDH/PDA#, and Zn-2# samples after femur was implanted for 8 weeks (c). Red triangles indicate the implant, yellow square indicates the new bone, and purple arrows indicate the corrosion layer.

For the study of *in vivo* osteogenesis ability, the rats were scarified and analyzed after implantation for 8 weeks. Figure 9a shows the transverse and longitudinal section views of micro-CT imaging. It can be seen that all the implants retained a complete shape, indicating their favorable corrosion resistance *in vivo*. The animal study by Yoshizawa et al. [53] also revealed that AZ31 experienced only a degradation volume of 0.5 mm^3^ after implanted for 8 weeks. The reconstructed 3D micro-CT images are shown in Fig. 9b. The new formed bone was marked in red. Larger scale of red areas was observed on the surface of LDH#, LDH/PDA#, and Zn-2# implants than that on AZ31 implant. Furthermore, the calculated BV/TV and BMD values are shown in Fig. 9c and d. No differences of BMD values were observed between all the four implants. The BV/TV values of LDH/PDA and Zn-2# samples were significantly higher than that of AZ31 and LDH# samples, indicating the better osteogenesis of the two PDA-coated samples. To investigate the osteointegration situation between the implants and the new bone, VG staining and SEM observation were carried out and the results are shown in Fig. 10a and b. For AZ31 and LDH# groups, scattered new bone grew in the bone marrow cavity, and the surfaces were covered by a layer of corrosion products (Fig. 10a). For LDH/PDA# and Zn-2# groups, although their surfaces were also covered with corrosion product, but more new bones were observed in the marrow cavity. More importantly, already formed new bone was closely surrounding the implant surfaces, especially for Zn-2# group. In Fig. 10b, the red triangles indicate the implant, yellow square indicates the new bone, and purple arrows indicate the corrosion layer. Corrosion layers were observed for all the implants. A continuous layer of new bone was observed surrounding Zn-2# implant and was closer to the implant. The SEM observation further confirmed the results of VG staining. The above data suggested the favorable *in vivo* osteogenesis and osteointegration of Zn-2# sample.

**Figure 11.**
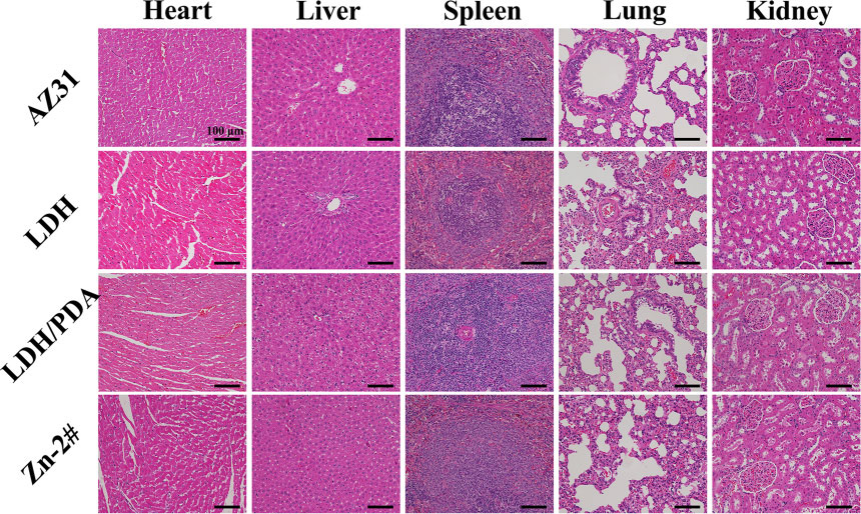
Typical histological morphology of important organic tissues in H&E sections of AZ31, LDH#, LDH/PDA#, and Zn-2# samples after femur implantation for 8 weeks.

Biosafety is also a concerned issue for bone implants. The corrosion products (such as Mg^2+^, OH^−^ and hydrogen) of Mg-based orthopedic devices would enter bloodstream, and then might damage organic tissues [54]. In this study, the major organs (including heart, liver, spleen, lung, and kidney) were harvested after the samples were implanted for 8 weeks, and stained with H&E staining (Fig. 11). For all the tested organic, no pathological changes were observed, indicating the suitable biosafety of all the implants.

## Conclusion

In this study, Zn-contained PDA film was successfully prepared on LDH-coated AZ31 surface via the self-polymerization process of dopamine. The as-prepared film exhibited stable Zn ion release and enhanced corrosion resistance.

With enhanced corrosion resistance, the film possessed a hemolysis ratio below 1% and a good cytocompatibility to MC3T3-E1 and RAW264.7. Owing to release of Zn ions, the film showed a strong antibacterial ability against *S. aureus*. In addition, the results of *in vitro* cellular response indicated that Zn-contained PDA film endow the materials with the capability of inducing macrophages polarize to M2 phenotype (anti-inflammation type) and promote osteogenic differentiation of MC3T3-E1. A femoral osteomyelitis model was applied to verify the *in vivo* anti-infection and pro-osseointegration abilities of the Zn-contained PDA film. In summary, with desirable antibacterial, anti-inflammation, and pro-osteointegration properties, the novel designed Zn-contained PDA-modified Mg alloys have large potential in orthopedic applications.

## Supplementary data

Supplementary data are available at *REGBIO* online.

## Funding

This study was supported by the National Key Research and Development Project (2017YFB0702600, 2016YFB0700800), China Postdoctoral Science Foundation (2019M662830), National Natural Science Foundation of China (51901239), Natural Science Foundation of Guangdong Province, China (Grant No. 2020A1515011447), The Joint Fund of Ministry of Education for Equipment Pre-research (No. 6141A02022632) and Scientific and Technological Projects of Guangzhou, China (Grant No. 202002030283).

## Supplementary Material

rbaa044_Supplementary_Data
