# Supplementary material for: Zn-contained mussel-inspired film on Mg alloy for inhibiting bacterial infection and promoting bone regeneration
Source: Regen Biomater. 2020 Sep 30;8(1):rbaa044. doi: 10.1093/rb/rbaa044 (PMC7947588; doi:10.1093/rb/rbaa044)
Supplement: rbaa044_Supplementary_Data [file rbaa044_supplementary_data.docx]

**Supporting Information**

**Zn-contained mussel-inspired film on Mg alloy for inhibiting bacterial infection and promoting bone regeneration**

Feng Peng^a,1^, Shi Cheng^a,1^, Ruiying Zhang^a,1^, Mei Li^a^, Jielong Zhou^a^, Donghui Wang^b,*^, Yu Zhang^a,*^

*^a^Department of Orthopedics, Guangdong Provincial People's Hospital, Guangdong Academy of Medical Sciences，Guangzhou, Guangdong, 510080, China*

*^b^School of Materials Science and Engineering, Hebei University of Technology, Tianjin 300130, China*

**^*^Corresponding Author:**

E-mail addresses: [wdh_81@163.com](mailto:wdh_81@163.com) (Donghui Wang), [luck_2001@126.com](mailto:luck_2001@126.com) (Y Zhang).

**^1^These authors contributed equally to this work.**


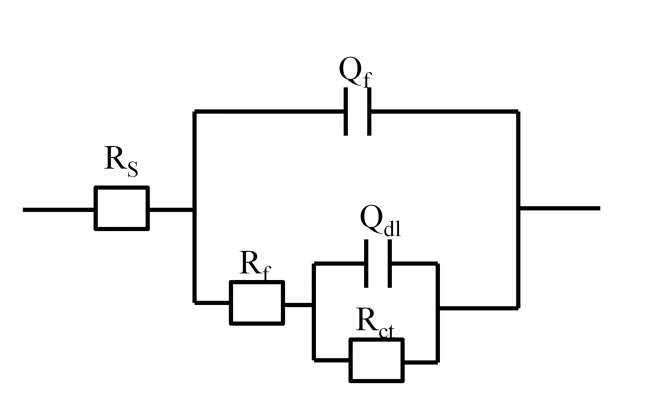


**Figure S1**. Equivalent circuit of EIS fitting for AZ31, LDH#, LDH/PDA#, and Zn-2# samples. *R_s_* is the solution resistance. *Q_f_* and *R_f_* are the capacitance and resistance of the corrosion production or coating on Mg, respectively. *R_ct_* and *Q_dl_* are the charge transfer resistance and the constant phase element of the electrical double layer, respectively.


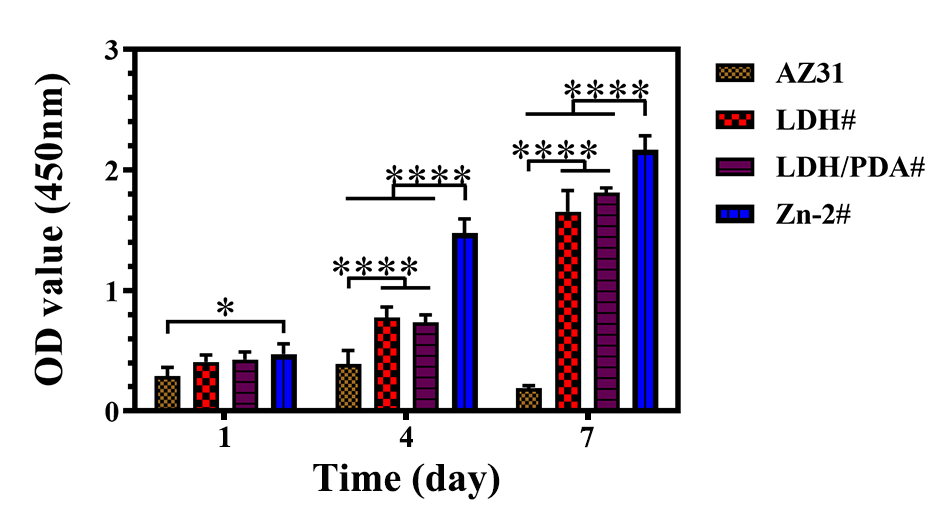


**Figure S2.** Cell proliferation of MC3T3-E1 after being cultured in the extracts of AZ31, LDH#, LDH/PDA# and Zn-2# (a)


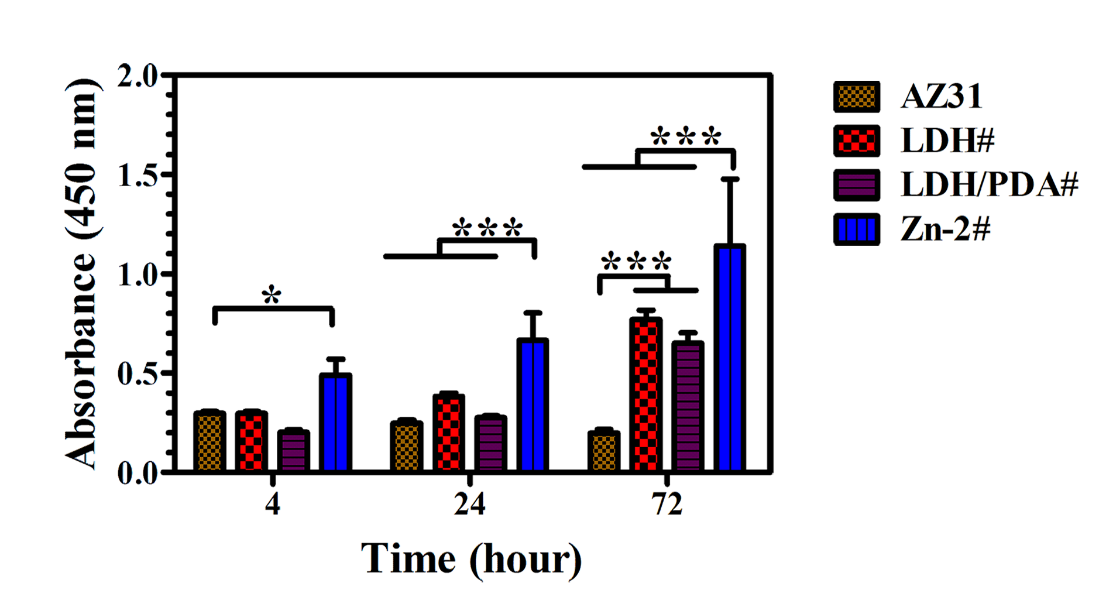


**Figure S3.** Cell proliferation of RAW264.7 cells after being cultured in the extracts of AZ31, LDH#, LDH/PDA# and Zn-2# (a)
